# Value of Plasma NGAL and Creatinine on First Day of Admission in the Diagnosis of Cardiorenal Syndrome Type 1

**DOI:** 10.1155/2020/2789410

**Published:** 2020-10-06

**Authors:** Hao Phan Thai, Bao Hoang Bui, Tien Hoang Anh, Minh Huynh Van

**Affiliations:** ^1^Department of Internal Medicine, Pham Ngoc Thach University of Medicine, Ho Chi Minh City, Vietnam; ^2^Hue University of Medicine and Pharmacy, Hue University, Hue, Vietnam; ^**3**^ Department of Internal Medicine, Hue University of Medicine and Pharmacy, Hue University, Hue, Vietnam

## Abstract

**Background:**

The presence of acute kidney injury in the setting of acute heart failure (AHF) or acute decompensated heart failure (ADHF) is a very common occurrence and was termed cardiorenal syndrome 1 (CRS1). Neutrophil gelatinase-associated lipocalin (NGAL) in the blood and urine is one of the earliest biomarkers of acute kidney injury due to ischemia or renal toxicity. This study was aimed to evaluate the diagnostic efficacy of plasma NGAL in the diagnosis of CRS1.

**Methods:**

There were 139 patients with AHF or ADHF in the department of Cardiovascular Resuscitation and Interventional Cardiology at Ho Chi Minh City 115 People Hospital from September 2018 to March 2019. This was a prospective cohort study.

**Results:**

There were 48 cases (rate 34.5%) with CRS1, mean age was 66.12 ± 15.77 and men accounted for 50.4%. There were no significant differences of vital signs at admission, diagnosis, and EF-based heart failure between CRS1 and non-CRS1 groups. The urea, creatinine on first day (creatinine D1) and third day (creatinine D3), NT-proBNP, and NGAL levels were higher in the CRS1 group than the non-CRS1 group, *p* < 0.05. The optimal cutoff plasma NGAL for diagnosing CRS1 was >353.23 ng/ml, area under curve (AUC) 0.732 (95% CI 0.65–0.80, *p* < 0.001), sensitivity 74.47%, specificity 68.48%, positive predictive value 54.7%, and negative predictive value 84%. Multivariable logistic regression analysis eGFR_CKDEPID1_ remained the strongest independent predictor of CRS1. Building the optimal regression model (without eGFRCKDEPID1) by the BMA (Bayesian model average) method with two variables NGAL and Creatinine D1, we had the equation: odds ratio = e_*y*_ while y = −2.39 + 0.0037 × NGAL + 0.17 × Creatinine D1. The nomogram (without eGFR_CKDEPID1_) was designed to predict the likelihood of CRS1 with AUC 0.79.

**Conclusions:**

The combination of plasma NGAL and creatinine D1 on the first day at admission had a high accuracy of predictive model for CRS1.

## 1. Introduction

### 1.1. Background

Acute kidney injury (AKI) in the setting of acute heart failure (AHF) or acute decompensated heart failure (ADHF) is a very common occurrence and was termed cardiorenal syndrome type 1 (CRS1) [1]. CRS is a disorder of the heart and kidneys that can cause acute or chronic dysfunction of one organ to cause another. CRS was divided into 5 types, of which the first type is called acute cardiorenal syndrome, which is an acute cardiac dysfunction leading to injury and/or acute renal dysfunction. The prevalence of cardiorenal syndrome type 1 according to studies varies from 32% to 40% in patients hospitalized for episodes of ADHF [2]. It is estimated that in the United States, there will be 320,000 to 400,000 hospitalizations with CRS type 1 every year. Moreover, with the increasing number of heart failure patients, the rate of CRS type 1 will be an important issue in the future.

In the CRS type 1, the diagnosis of acute kidney damage is often delayed because of the creatinine and urine output according to KDIGO (Kidney Disease Improving Global Outcomes). Neutrophil gelatinase-associated lipocalin (NGAL) in the blood and urine is one of the earliest indicators of acute kidney injury due to ischemia or nephrotoxicity. One study showed using NGAL in urine to diagnose acute kidney injury with 90% sensitivity and 99% specificity [3]. Neutrophil gelatinase-associated lipocalin (NGAL), a protein of the lipocalin superfamily, is synthesized abundantly in kidney tubules. Its expression is rapidly upregulated by ischemia-reperfusion injury in renal tubular epithelial cells, and NGAL is released into urine in an experimental model. In humans, NGAL has been recognized as a surrogate marker of AKI complicated with various diseases, including sepsis, postcardiac surgery, and admission to the intensive care unit. In particular, a few studies reported an association between the elevation of serum NGAL levels on admission and consequent AKI in patients with chronic heart failure [3]. However, in AHF or ADHF patients, the diagnostic value of plasma NGAL for CRS1 remains poorly understood.

### 1.2. Study Objectives

We aimed to evaluate the diagnostic efficacy of NGAL in diagnosis of CRS1 in AHF or ADHF patients.

## 2. Materials and Methods

### 2.1. Selection of Participants

All patients with AHF or ADHF were admitted to the Cardiovascular Resuscitation and Interventional Cardiology Department of 115 People Hospital in Ho Chi Minh City from November 2018 to May 2019.

Inclusion criteria for this study were adult inpatients (≥18 years old) with AHF or ADHF with or without CRS type 1.

Criteria for diagnosing AHF or ADHF was used according to Canadian Cardiovascular Society guidelines for the management of heart failure 2017 [4].

  Criteria for diagnosing AKI: according to the KDIGO clinical practice guideline for acute kidney injury 2012 [5], serum creatinine increased ≥0,3 mg/dL (≥26.5 *µ* mol/l); or within 48 hours, there was a 50% increase in serum creatinine from the level on admission during hospitalization. Urine criteria (0.5 mL/kg per hour for 6 hours) were not utilized for AKI diagnosis because of the potential alterations in the urine volume induced by therapeutic medication.
  Criteria for diagnosing CRS type 1: patients who suffered from AHF or ADHF developed AKI within 48 hours [6].

Exclusion criteria were not agreeable to participation; hospitalization period <2 days; multiple organ failure or septic shock; AKI caused by contrast; renal dialysis; kidney transplant; progressive hepatitis; acute pancreatitis; long-term use of high dose steroids; cyclosporin; and malignancy.

### 2.2. Study Design and Sample Size

This was a prospective cohort study.

#### 2.2.1. Sample Size

This was a diagnostic study; the sample size is calculated by the Buderer formula [7]:

(1) $$\begin{array}{c} n_{se}=\frac{Z_{\alpha }^{2}\times P_{se}\times \left(1-P_{se}\right)}{W^{2}\times P_{\text{dis}}},\\ n_{sp}=\frac{Z_{\alpha }^{2}\times P_{sp}\times \left(1-P_{sp}\right)}{W^{2}\times \left(1-P_{\text{dis}}\right)}, \end{array}$$

where *n*_*se*_ estimated the sample size to estimate for sensitivity, *n*_*sp*_ estimated the sample size to estimate for specificity, and *P*_*se*_ is the reference sensitivity according to the literature. For NGAL, this sensitivity is 100% [8]; *P*_*sp*_ is the reference specificity according to the literature. For NGAL, this specificity is equal to 86.7% [8]; *P*_dis_ is the rate of CRS type 1 according to Fabbian et al. which is 48.2% [9]; Z is the constant of the normal distribution, with a type I error of 5%; we have *Z*_*α*_^2^ = 1.96; W^2^ is the true positive and true negative error of the 95% confidence interval; we chose *W* = 0.15.

The required sample size *n* only needed to be larger than *n*_*se*_ and *n*_*sp*._ For NGAL, we calculated *n*_se_ = 31.9 and *n*_sp_ = 38. So, *n* ≥38 patients. Therefore, the minimum sample size would be 38 patients.

### 2.3. Clinical Evaluation and Biomarker Measurements

All patients were taken medical history; meticulous physical examination; assessment of vital signs such as pulse and systolic and diastolic blood pressure; jugular venous distention, S3, murmurs, rales, and edema. It was then tested: first day serum creatinine (creatinine D1) and third day serum creatinine (creatinine D3) with Alinity c creatinine reagent running on Abbott's Alinity machine; plasma NGAL with human NGAL ELISA kit 036RUO of BioPorto Diagnostics A/S Copenhagen, Denmark; NT-proBNP with the Elecsys® proBNP II reagent kit from Roche Diagnostics, Bromma, Sweden, running on a Cobas e411 analyzer; these tests were performed at the laboratory department of Medic Medical Center 254 Hoa Hao Street, District 10, Ho Chi Minh City, Vietnam. Additional tests cell blood counts, urea, AST, ALT, electrolytes panel, and arterial blood gas were performed at the laboratory department of 115 People Hospital. Electrocardiography, chest X-ray, echocardiography, medications on admission and follow-up during hospital stay : length of hospital stay and in-hospital mortality, or serious illness were recorded. The estimated glomerular filtration rate was calculated using the 2009 CKD-EPI creatinine formula (eGFR_CKDEPI_).

### 2.4. Statistical Analysis

Data were processed using IBM SPSS Statistics Version 25 software, MedCalc @ version 19.0.5 software, and RStudio version 1.2.5001 software (http://www.rstudio.com). A statistical significance level of 0.05 was used. All hypothesis testing were two-tailed. Discrete variables are expressed as counts (percentage) and continuous variables as mean ± standard deviation (SD) or median and interquartile range (IQR). The means and rates of the two groups were compared by the *t*-test and the chi-square test; the ROC curve was used to calculate the AUC. The cutoff value was chosen at the highest score of Youden (J) with J = sensitivity + specificity − 1. Sensitivity, specificity, positive predictive value (PPV), and negative predictive value (NPV) were calculated.

The correlation between two normally distributed continuous variables was evaluated by Pearson; otherwise, Spearman correlation was used. Binary univariate logistic regression analysis between CRS1 and variables was performed. The variables with *p* value <0.1 were selected in the multivariate logistic regression model by the Wald test with backward-stepwise method to determine predictors.

Optimal regression models were determined by the Bayesian model average method; the most optimal model was chosen with the smallest BIC (Bayesian information criteria) and the highest postprobabilities [9].

A forecasting model was built by dividing data into two small datasets: “training set” accounting for 60% of data and “testing set” accounting for 40% of data. A forecasting model in a “training set” was developed, and then the forecasting model on a “testing set” using 10-fold cross-validation was validated to evaluate the model in the “testing set”.

Finally, the accuracy, sensitivity, specificity, positive predictive value, and negative predictive value were calculated by confusion matrix. The forecasting model was represented by (1) forecasting equation; (2) nomogram; and (3) dynamic nomogram posted on the website.

The study was carried out according to the principles of the Declaration of Helsinki. It was approved by the local ethical committee. Written informed consent was obtained from all participants.

## 3. Results

During November 2018 and May 2019, 172 patients were initially diagnosed with AHF or ADHF. After follow-up, 33 cases were excluded from the study because they did not meet the inclusion criteria; we eventually collected 139 cases of AHF or ADHF that met inclusion criteria and no exclusion criteria. Among 139 cases, there were 48 cases of diagnosis of CRS type 1 accounting for 34.5%. Data were divided into two groups with CRS type 1 (CRS1, *n* = 48) and no CRS type 1 (non-CRS1, *n* = 91). In the CRS1 group, there were 04 cases without EF evaluation and 01 case without cell blood count; the non-CRS1 group had 07 cases without evaluation of EF and 01 case without cell blood count.

### 3.1. Demographic and Clinical Characteristics

Detailed baseline characteristics of the study population are summarized in Table 1. The mean age of the patients was 66.12 ± 15.77, minimum 20 years old and maximum 96 years old. Male/female ratio was 1.01; BMI, median, and interquartiles of the two groups were 23.44 [21.56–25.05], statistically significant difference at *p* < 0.05.

The majority of patients with a history of arterial hypertension accounted for 63.3%, followed by medical history of diabetes 36.7%, heart failure 32.6%, and chronic kidney disease 15.8%. There were no differences in medical history between two groups with CRS1 and non-CRS1, *p* > 0.05. However, there was a difference in the patients with Hx chronic kidney disease between the two groups, statistically significant at *p* < 0.05.

There were 60 cases (43.2%) which were diagnosed with acute pulmonary edema; 38.8% were ADHF; 16.5% were cardiogenic shock; 56 patients (40.9%) had acute myocardial infarction. There were 65 cases (50.8%) of heart failure with preserved EF ≥50%; 26.6% with EF <40% reduced heart failure; 22.7% with 40–49% midrange EF heart failure. There was no difference in vital signs at admission, diagnosis, and type of EF-based heart failure between two groups, *p* > 0.05.

There were similarities in laboratory values at admission: neutrophil, hemoglobin, liver enzymes (AST, ALT), troponin I, arterial blood gases (pH, HCO_3_^−^, pCO_2_, pO_2_), Na^+^, and K^+^ concentration between the two groups. However, the concentrations of urea, creatinine D1 and D3, plasma NGAL, and NT-proBNP in the CRS1 group were higher than those in the non-CRS1 group; the differences were statistically significant at *p* < 0.05. eGFR by creatinine on the first day (eGFR_CKDEPID1_) and third day (eGFR_CKDEPID3_) in the CRS1group was lower than that in the non-CRS1group, *p* < 0.05.

The majority of patients using furosemide diuretics accounted for 77.7%, the mean dose was 40 mg. Nitrates were used in 85 patients (61.2%). Only one patient (0.7%) used beta-blockers, up to 18.7% received noradrenaline. There were 2 patients (1.4%) who indicated continuous renal replacement therapy in the CRS1 group, but the differences between two groups were not statistically significant at *p* > 0.05. There were similarities in treatment at admission between the two groups.

The length of hospital stay of the two groups with the median was 9 days, the interquartile was 7–12 days. Length of hospital stay in the CRS1 group was longer than that in the non-CRS1 group, but this difference was not statistically significant at *p* > 0.05. In-hospital mortality or serious illness was 21 cases, accounting for 15.1%. In-hospital mortality/serious illness was higher in the CRS1 group compared with the non-CRS1 group, *p* < 0.05.

### 3.2. The Value of Plasma NGAL in Diagnosing CRS1

**Figure 1:**
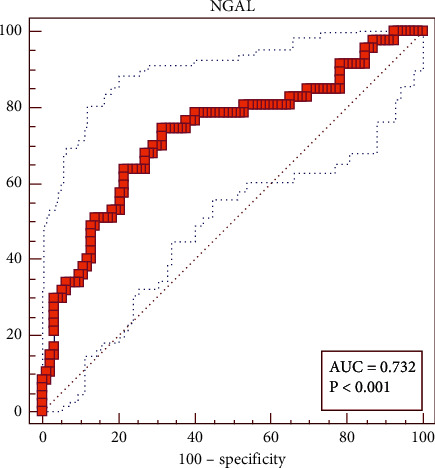
Cutoff point, sensitivity, and specificity of plasma NGAL for diagnosing CRS1.

The diagnostic accuracy of the NGAL was evaluated using the receiver operating characteristic (ROC) curve analysis. The optimal cutoff point of NGAL to diagnose CRS1 was >353.23 ng/ml, the area under the AUC curve 0.732 (95% CI 0.65–0.80, *p* < 0.001), sensitivity 74.47%, specificity 68.48%, positive predictive value 54.7%, and negative predictive value 84%. The result was displayed in Table 2 and Figure 1.

### 3.3. The Correlation between CRS1 and Some Factors

To investigate the correlation between CRS1 and several factors, we conducted Pearson correlation analysis if variables were in normal distribution; otherwise, Spearman rank was used. As a result, there were six variables correlating to CRS1 as in Table 3.

### 3.4. Univariable and Multivariable Logistic Regression between CRS1 and Some Variables

Six variables correlated with CRS1 were analysed by univariable logistic regression. The variables with *p* value <0.1 were selected in the multivariate logistic regression model by the Wald test with backward-stepwise method. During multivariable regression analysis, eGFR_CKDEPID1_ remained the strongest independent predictor of CRS1 (OR 0.96; 95% CI 0.94–0.98; *p* < 0.001). Plasma NGAL failed to predict the occurrence of early CRS1. The result is presented in Table 4.

### 3.5. The Optimal Multivariate Model for Correlation between CRS1 and Some Factors by BMA (Bayesian Model Average) Method

**Figure 2:**
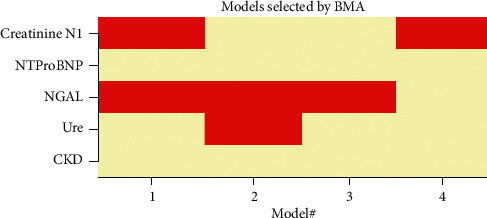
Models were selected by the BMA method.

According to modern statistical theory, in order to find the optimal model of correlation between CRS1 and some factors, we used the BMA model [10] using *R* software to process. Variables likely to predict CRS1, including creatinine D1, eGFR_CKDEPI1_, urea, NT-proBNP, NGAL, and history of chronic kidney disease, were entered in the regression equation for processing. Because eGFR_CKDEPI_ was calculated by creatinine, we did not enter simultaneously these two variables in regression equation because of according to regression theory; variables must be independent in the multivariate regression equation. The results selected four most optimal models with cumulative posterior probability = 1 (Figure 2) (without eGFRCKDEPI) and one model only contained eGFRCKDEPI (with eGFRCKDEPI).

Based on the results of selecting the optimal regression models according to the BMA method, there are four models in which model 1 was with the smallest BIC (−4.97e^+02^ = −36.72) and highest post probability (0.274 = 27.4%) was considered the most optimal model with 2 variables: creatinine D1 and plasma NGAL correlation with CRS1.

### 3.6. Building Forecasting Model for CRS1 with NGAL and Creatinine D1

Building forecasting model by dividing data into two small datasets: training set accounting for 60% of data and testing set accounting for 40% of data. Developing a forecasting model in a “training set”:

(2) $$\begin{array}{c} \text{Odds ratio}=e^{y}\text{,}\;\text{where }y=-2.39+0.0037\times \text{NGAL}+0.17\times \text{CreatininD}1. \end{array}$$

Then, the forecasting model on a “testing set” using 10-fold cross-validation was validated to evaluate the model in the “testing set” by methods with accuracy 79.82%; kappa = 0.54; AUC = 0.79; accuracy 75.93%; sensitivity 76.74%; specificity 72.73%; positive predictive value 91.67%; and negative predictive value 44.44% by confusion matrix (Table 5).

### 3.7. Nomogram in Predicting CRS1 with 2 Variables NGAL and Creatinine D1 and Model Visualisation by Dynamic Nomogram

#### 3.7.1. Clinical Application of the Nomogram with Plasma NGAL and Creatinine D1

**Figure 3:**
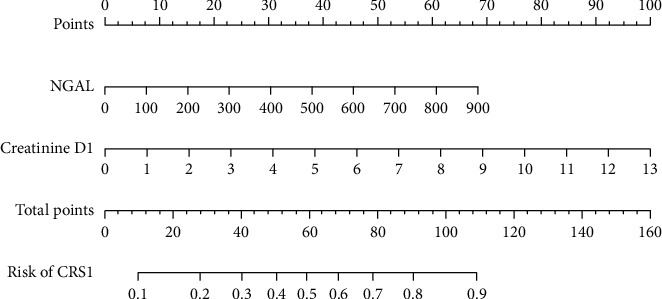
Nomogram in predicting CRS1 of plasma NGAL and creatinine D1.

**Figure 4:**
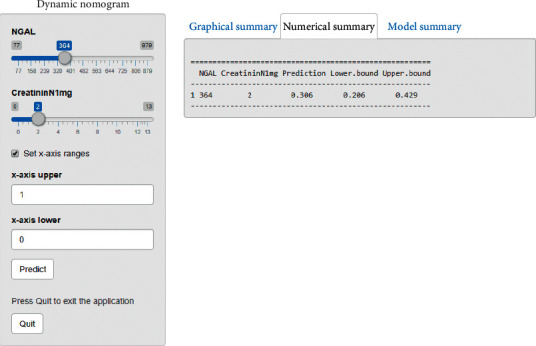
Dynamic nomogram in predicting CRS1 of plasma NGAL and creatinine D1.

Nomogram in predicting CRS1 with the variables NGAL and creatinine D1 is shown in Figure 3 and model visualisation by dynamic nomogram is shown in Figure 4 (http://127.0.0.1:3540).

2 cases were taken to illustrate the application of the nomogram in predicting CRS1.

First case had a creatinine concentration of 4.59 mg/dl on the first day corresponding to 35 points and the plasma NGAL concentration on day 1 was 631.71 ng/ml corresponding to 47 points. The total score was 35 + 47 = 82 points, the probability of having CRS1 is more than 70%. In fact, this patient was diagnosed with CRS1.

The second case had a creatinine concentration of 0.87 mg/dl on the first day corresponding to 5 points and the plasma NGAL concentration on day 1 was 222.25 ng/ml corresponding to 7 points. The total score was 5 + 7 = 12 points; the probability of developing CRS1 was less than 10%. The risk of CRS1 is very low. In fact, this patient did not suffer from CRS1.

## 4. Discussion

In this study, the mean age of patients was 66.12 ± 15.77. The percentage of female patients with AHF or ADHF in our study was 49.6%. When compared with previous studies, our results are similar to those in [3, 11–14]; in contrast, the male rate in our study was lower than that of Aghel et al. [15].

Tachycardia at admission with a median was 102 beats/minute and interquartile range was [88–114]. While systolic blood pressure was 120 [90–140] mmHg and diastolic blood pressure was 70 [60–80] mmHg. Percentage of patients diagnosed with acute pulmonary edema, ADHF, and cardiogenic shock were 43.2%, 38.8%, and 16.5%, respectively. There were 56 patients (40.9%) diagnosed with acute myocardial infarction. Only 5 patients (5/56 = 8,9%) with acute myocardial infraction had percutaneous coronary intervention and all patients were tested NGAL, cystatin C, and NT-proBNP before percutaneous coronary intervention. There are similarities in vital signs at admission and diagnosis between two groups with CRS1 and non-CRS1. This was also explained by the fact that both groups were patients with AHF or ADHF.

The present study revealed a similarity in laboratory values at admission: left ventricular ejection fraction EF, neutrophil, and hemoglobin between the two groups CRS1 and non-CRS1. However, urea concentration, creatinine D1 and D3, NT-proBNP, and NGAL in the CRS1 group were higher than the non-CRS1 group. Conversely, sodium concentration, eGFR_CKDEPID1_, and eGFR_CKDEPID3_ in the CRS1 group were lower than the non-CRS 1 group, *p* < 0.05. Patients in the CRS1 group had considerably lower GFR and higher creatinine and NGAL at baseline. One possible explanation is the rate of history of chronic kidney disease in the CRS1 group being higher than the non-CRS1group, as NGAL and cystatin C have been shown to be elevated in patients with chronic kidney disease. Unfortunately, these findings made cutoff points for diagnosing CRS1 of NGAL which were higher than patients without history of chronic kidney disease, but this was unavoidable clinically because chronic kidney disease is a comorbidity in heart failure.

Neutrophil gelatinase-associated lipocalin (NGAL), a protein of the lipocalin superfamily, is synthesized abundantly in kidney tubules. Its expression is rapidly upregulated by ischemia-reperfusion injury in renal tubular epithelial cells, and NGAL is released via urine in an experimental model [3]. In humans, NGAL has been recognized as a surrogate marker of AKI complicated with various diseases, including sepsis, postcardiac surgery, and admission to the intensive care unit [3]. In this study, plasma NGAL concentrations in the CRS1 group 506.49 [322.51–591.80] ng/ml was higher than that in the non-CRS1 group 1263.89 [193.07–409.46] ng/ml, *p* < 0.001. The cutoff point was >353.23 ng/ml, AUC curve 0.732 (95% CI 0.65–0.80, *p* < 0.001), sensitivity 74.47%, specificity 68.48%, positive predictive value 54.7%, and negative predicted value 84%. The plasma NGAL concentration and cutoff point for the diagnosis of CRS1 in our study was higher than that of the author Margarida et al. [13]. This might be explained by the different NGAL test kit, so the results will be different.

When entering the variables into a univariate logistic regression analysis, 6 variables predicted the occurrence of early CRS1. During multivariable regression analysis, eGFR_CKDEPID1_ remained the strongest independent predictor of CRS1. Plasma NGAL failed to predict the occurrence of early CRS1. In case of without eGFR_CKDEPID1_, there were two variables (creatinine D1 and NGAL) independently predicting CRS1.

We built a predictive model for CRS1 with two variables, plasma NGAL and creatinine D1, by the equation: Odds ratio *e*^*y*^, where *y* = −2.39 + 0.0037 × NGAL + 0.17 × creatinine D1 and nomogram, as shown in Figure 3. With the plasma NGAL concentration on the 1st day, we will have the corresponding score and creatinine D1 concentration will have the corresponding score and the total score will correspond to the risk of CRS1. This result was different from the research results of Zeyuan Fan et al.; the nomogram includes 6 variables: age, diabetes mellitus, hsCRP, eGFR, NYHA, and urine albumin/creatinine ratio [16]. The reason for this difference is that in our research, when building the optimal regression model, only two variables NGAL and creatinine were the best models.

Several reports evidenced the role of NGAL in the setting of heart failure, underlying the leading part of tubular damage independently from baseline renal dysfunction [17–19]. Data from the Gruppo Italiano per lo Studio della Sopravvivenza nell'Insufficienza Cardiaca (GISSI-HF) trial showed that urinary NGAL is a good marker for tubular damage, and it is significantly related to adverse outcomes in patients with chronic heart failure and preserved renal function [20]. In acute heart failure, data are less clear: Legrand et al. [21] were not able to demonstrate a diagnostic role of urinary NGAL to early recognize renal injury, whereas Aghel et al. [15] revealed that admission serum NGAL levels (140 ng/ml) had a 7.4-fold increase in the risk of developing renal dysfunction; high levels are also related to poor outcome in a follow-up period.

### 4.1. Study Limitations

There are several limitations to the study. First, this study was conducted in a single center in Vietnam, limiting the external validity to other centers with different settings. Second, most patients are seriously ill so they have not been fully assessed for hospitalization because ADHF patients may not be admitted to the cardiac resuscitation department. Third, some kidney diseases (such as urinary tract infections or immune diseases) can also lead to an increase in NGAL levels. Although we had tried to eliminate these patients with a history and physical examination, they were still not completely controlled. Fourth, we did not measure hemodynamics or more accurate measurements of glomerular filtration rate to directly link the increased NGAL level to the compensatory kidney condition. Fifth, our sample size is still relatively small and there were some missing data. Sixth, we only evaluated for CRS1 within 48 hours, so we can skip cases with CRS1 after 48 hours to 7 days. Lastly, we only tested plasma NGAL once in the first day but did not test after 48 hours and before discharge to assess the variability of plasma NGAL concentration compared with creatinine concentration.

## 5. Conclusion

Plasma NGAL was valuable for the diagnosis of CRS1 with a cutoff point >353.23 ng/ml, the AUC curve was 0.732 (95% CI 0.65–0.80, *p* < 0.001), sensitivity 74.47%, specificity 68.48%, positive predictive value 54.7%, and negative predictive value 84%. The predictive model of CRS1, including 2 variables plasma NGAL and creatinine D1, had accuracy of 75.93%, sensitivity 76.74%, specificity 72.73%, positive predictive value 91.67%, and negative predictive value 44.44%.

## Figures and Tables

**Table 1:** Demographic and clinical characteristics.

Variable	Total (*n* = 139)	CRS1 (*n* = 48)	Non-CRS1 (*n* = 91)	*p* value
Age (years)	66.12 ± 15.77	64.06 ± 15.29	67.19 ± 15.98	0.27
Male	70 (50.4)	24 (51.4)	46 (50)	0.95
Body mass index^*∗*^ (kg/m^2^)	**23.44 [21.56–25.05]**	**24.29 [22.5–25.82]**	**23.44 [21.33–24.38]**	**0.037**
Medical history				
Arterial hypertension	88 (63.3)	34 (70.8)	54 (59.3)	0.18
Diabetes mellitus	51 (36.7)	20 (42.6)	31 (33.7)	0.38
Dyslipidemia	9 (6.5)	4 (8.5)	5 (5.4)	0.49
Smoking	14 (10.1)	5 (10.4)	9 (9.9)	0.92
Alcohol drinking	1 (0.7)	1 (2.1)	0 (0)	0.17
IHD/old MI	42 (30.2)	15 (31.3)	27 (29.7)	0.85
DCM	5 (3.6)	2 (4.2)	3 (3.3)	0.56
Valve heart diseases	25 (18)	5 (10.4)	20 (21.9)	0.092
Heart failure	45 (32.6)	17 (35.4)	28 (30.8)	0.61
CKD	**22 (15.8)**	**12 (25)**	**10 (10.9)**	**0.031**
Stroke	10 (7.2)	4 (8.3)	6 (6.6)	0.74
Vital signs at admission				
Heart rate (beats/min)	102 [88–114]	98 [84–115]	104 [90–114]	0.89
BP (mmHg)				
Systolic	120 [90–140]	120 [90–140]	110 [100–140]	0.79
Diastolic	70 [60–80]	70 [60–80]	70 [60–80]	0.29
Mean	86.67 [70–100]	86.67 [70–100]	86.67 [73.33–100]	0.58
Oxygen saturation (%)^*∗∗*^	90 [86–95]	90 [87–96]	90 [86–94]	0.53
Diagnosis				
APE	60 (43.2)	15 (31.3)	45 (49.5)	0.11
Cardiogenic shock	23 (16.5)	9 (18.8)	14 (15.4)	
ADHF	54 (38.8)	24 (50)	30 (32.9)	
Others	2 (1.4)	0 (0)	2 (2.2)	0.66
Acute MI	56 (40.9)	18 (37.5)	38 (41.8)	
Laboratory values				
EF^*∗∗∗*^-based HF				
EF reduced	34 (26.6)	9 (20.9)	25 (29.4)	0.29
EF midrange	29 (22.7)	13 (30.2)	16 (18.8)	
EF preserved	65 (50.8)	21 (48.8)	44 (51.8)	
Neutrophil^#^ (K/*µ*L)	7.84 [5.50–10.71]	8.5 [5.37–11.96]	7.73 [5.50–10.32]	0.39
Hb (g/dl)^#^	11.60 [9.98–13.53]	10.8 [9.13–13.38]	12.15 [10.4–13.60]	0.087
AST (UI/l)^##^	47.49 [28.98–104.83]	48.2 [30.2–106.33]	46.9 [28.58–104.83]	0.41
ALT (UI/l)^##^	29.7 [17.86–79.04]	33.11 [17.78–85.64]	28.02 [18.08–69.20]	0.94
Urea (mmol/l)^###^	**9.82 [6.20–14.53]**	**12.67 [8.51–19.27]**	**8.09 [5.45**–**11.67]**	**<0.01**
Creatinine D1(mg/dl)	**1.31 [0.99–2.24]**	**2.44 [1.47–4.09]**	**1.08 [0.83–1.47]**	**<0.01**
eGFR_CKDEPID1_	**47 [23–75.75]**	**22 [13–44]**	**64 [38.25–84.05]**	**<0.01**
Creatinine D3	**1.29 [0.87–2.32]**	**2.84 [1.38–4.8]**	**1.07 [0.8–1.44]**	**<0.01**
eGFR_CKDEPID3_	**50 [23.25–79]**	**19.5 [11–47.5]**	**67 [38–86.50]**	**<0.01**
Na^+^ (mmol/l)	137.4 [133.48–140.48]	136.8 [130.55–138.8]	138.4 [135.03–141.05]	0.49
K^+^ (mmol/l)	4.05 [3.54–4.49]	4.15 [3.58–4.59]	3.96 [3.52–4.44]	0.54
NGAL (ng/ml)	**327.13 [205.38–516.76]**	**511.63 [338.27–587.94]**	**262.59 [193.07–401.11]**	**<0.001**
NT-proBNP (pg/ml)	**8814 [3860–26419]**	**20131 [6350–35000]**	**6378 [2935.25–17177.50]**	**0.005**
Troponin I^$^ (pg/ml)	6156.18 ± 13176.59	6575.08 ± 13505.34	5941.86 ± 13080.16	0.79
pH^$$^	7.40 ± 0.087	7.39 ± 0.099	7.42 ± 0.079	0.08
HCO_3_^−$$^ (mmol/l)	21.8 [17.85–24.98]	20.03 [16.4–23.7]	22.6 [19.1–25.98]	0.25
pCO_2_^$$^ (mmHg)	35 [29.08–40.03]	35 [27.85–40.95]	35 [29.98–39.48]	0.67
pO_2_^$$^ (mmHg)	76 [61.75–111]	75 [60–110.5]	77 [62.75–111]	0.77
Therapy at admission				
Furosemide	108 (77.7)	36 (75)	72 (79.1)	0.58
Furosemide dose (mg)	40 (20–40)	40 (20–40)	40 (20–40)	0.50
ACEIs/ARBs use	14 (10.1)	4 (8.3)	10 (10.98)	0.62
Beta-blockers	1 (0.7)	0 (0)	1 (1.1)	0.66
Dobutamine	19 (13.8)	7 (14.6)	12 (13.2)	0.84
Dopamine	7 (5)	3 (6.3)	4 (4.4)	0.64
Noradrenaline	26 (18.7)	9 (18.8)	17 (18.7)	0.99
Nitrates	85 (61.2)	28 (58.3)	57 (62.6)	0.62
Conventional oxygen	110 (79.1)	41(85.4)	69 (75.8)	0.19
Ventilation invasive	12 (8.6)	5 (10.4)	7 (7.7)	0.59
Mechanical ventilation	13 (9.4)	5 (10.4)	8 (8.8)	0.75
CRRT	2 (1.4)	2 (4.2)	0 (0)	0.051
Length of hospital stay (days)	9 [7–12]	10 [7–12]	8 [7–12.75]	0.33
In-hospital mortality/serious illness	**21 (15.1)**	**12 (25)**	**9 (9.9)**	**0.018**

Data are presented as *n* (%); medium ± SD; and median [interquartile range]. ^*∗*^*n* = 113; ^*∗∗*^*n* = 131; ^*∗∗∗*^*n* = 128;^#^*n* = 137; ^##^*n* = 115; ^###^*n* = 134; ^$^*n* = 130; ^$$^*n* = 117; EF reduced <40%; EF midrange 40–49%; EF preserved ≥50%. APE, acute pulmonary edema; BP, blood pressure; MI, myocardial infraction; IHD, ischemic heart disease; DCM, dilated cardiomyopathy; CCRT, continuous renal replacement therapy; ACEIs, angiotensionogen-converting enzyme inhibitors; ARBs, angiotensin II receptor blockers. Bold indicates statistical significance. Serious illness: high risk of mortality patients were resuscitated but their families asked to be discharged before death in the hospital.

**Table 2:** Cutoff point, sensitivity, specificity, and AUC of NGAL diagnosing CRS1.

Variable	Cutoff point	Sensitivity Se (%)	Specificity Sp (%)	Area under curve (AUC)	Confident interval (95% CI)	*p* value
NGAL (ng/ml)	>353.23	74.47	68.48	0.73	0.65–0.80	<0.001

**Table 3:** The correlation between CRS1 and some variables.

Variable	Coefficients of Pearson *r* or Spearman rho	*p* value
Age (years)	−0.11	0.20
Sex (male/female)	−0.01	0.91
Heart rate (beats/min)	−0.09	0.29
Systolic blood pressure (mmHg)	−0.032	0.71
Diastolic blood pressure (mmHg)	0.14	0.11
Mean blood pressure (mmHg)	0.004	0.96
Hb (g/dl)	−0.13	0.14
**Urea (mmol/l)**	**0.32**	**<0.001**
**Creatinine D1 (mg/dl)**	**0.38**	**<0.001**
**eGFR**_**CKDEPID1**_	−**0.48**	**<0.001**
**NGAL (ng/ml)**	**0.40**	**<0.001**
**NT-proBNP (pg/ml)**	**0.22**	**0.01**
**Hx of chronic kidney disease**	**0.19**	**0.025**
Hx of hypertension	0.10	0.23
Hx of diabetes mellitus	0.087	0.31
Hx of heart failure	0.022	0.79
Atrial fibrillation	−0.03	0.71

Bold indicates statistical significance. Hx, history.

**Table 4:** Univariable and multivariable logistic regression between CRS1 and some variables.

Univariable logistic regression				
Predictors	*β*	SE	Odds ratio (CI 95%)	*p* value

Urea (mmol/l)	0.10	0.031	1.11 (1.04–1.18)	0.001^*∗*^
Creatinine D1 (mg/dl)	0.63	0.16	1.87 (1.38–2.54)	<0.001^*∗*^
eGFR_CKDEPID1_	−0.045	0.009	0.96 (0.94–0.97)	<0.001^*∗*^
NGAL (ng/ml)	0.005	0.001	1.005 (1.0029–1.0074)	<0.001^*∗*^
NT-proBNP (pg/ml)	0.000	0.000	1.00	0.016^*∗*^
Hx CKD	1.034	0.47	2.81 (1.11–7.11)	0.029^*∗*^
Multivariable logistic regression				
Predictors	*β*	SE	Odds ratio (CI 95%)	*p* value
eGFR_CKDEPI_	−0.045	0.009	0.96 (0.94–0.98)	<0.001^*∗*^

Multivariable analysis included all significant candidate variables (*p* < 0.1) identified in univariate analysis. ^*∗*^*p* < 0.05.

**Table 5:** Confusion matrix in the model predicting CRS1.

Predicted outcome	Not present	Present
Predicted CRS1^−^	33 (true negative)	3
Predicted CRS^+^	10	8 (true positive)

## Data Availability

Data used for the current study are accessible on reasonable request from the corresponding author.
